# Procalcitonin as a potential tumor marker for fibrolamellar hepatocellular carcinoma: Insights from three patients and a literature review

**DOI:** 10.3389/fmed.2026.1718663

**Published:** 2026-02-02

**Authors:** İlgin Özden, Serhat Kaya, Türkan Dübüş, Ali Yücesan, Nilüfer Bulut, Emin Büyüktalancı

**Affiliations:** 1 Department of General Surgery (Hepatopancreatobiliary Surgery Unit), Başakşehir Çam & Sakura City Hospital, Istanbul, Türkiye; 2 Department of Radiology, Başakşehir Çam & Sakura City Hospital, Istanbul, Türkiye; 3 Department of Thoracic Surgery, Başakşehir Çam & Sakura City Hospital, Istanbul, Türkiye; 4 Department of Thoracic Surgery, Hamidiye Faculty of Medicine, University of Health Sciences, Istanbul, Türkiye; 5 Department of Orthopedic Surgery, Başakşehir Çam & Sakura City Hospital, Istanbul, Türkiye; 6 Department of Medical Oncology, Başakşehir Çam & Sakura City Hospital, Istanbul, Türkiye; 7 Department of Medical Oncology, Hamidiye Faculty of Medicine, University of Health Sciences, Istanbul, Türkiye; 8 Department of Pathology, Başakşehir Çam & Sakura City Hospital, Istanbul, Türkiye

**Keywords:** bone metastasis, fibrolamellar hepatocellular carcinoma, hemangioma, misdiagnosis, procalcitonin

## Abstract

Fibrolamellar hepatocellular carcinoma (fHCC) was diagnosed through magnetic resonance imaging in three young patients (with biopsies confirming the diagnosis in two of them prior to referral) aged 17, 22, and 24 years. All had normal or near-normal standard biochemistry results, except for increased levels of procalcitonin (PCT) and C-reactive protein (CRP) in the absence of any infectious source: PCT: 41, 5.8, and 1.2 ng/mL, respectively (normal range < 0.05 ng/mL) and CRP: 127, 21, and 14 mg/L, respectively (normal range: 0–5 mg/L). Right hepatectomy with negative surgical margins resulted in the eventual normalization of CRP levels in all patients, and PCT levels normalized in the latter two patients. The PCT level was 0.07 ng/mL at 3 months in the first patient. The emergence of two bone metastases in the rib and right femur at 7 months was accompanied by an increase in PCT levels to 0.8 ng/mL. Radiotherapy provided transient control in the femur only. Consequently, a thoracic wall resection was performed, followed by a segmental resection of the femur. Treatment with bevacizumab at a dose of 15 mg/kg/day and atezolizumab at 1200 mg/day every 21 days was started. A PET-CT scan conducted at the 42nd month showed no signs of recurrence, and the PCT level was 0.04 ng/mL. The other patients exhibited normal PCT levels and no evidence of recurrence at 16- and 6-months post-treatment. Since PCT measurement is routinely available, baseline levels should be measured at least once during the workup of patients with fHCC.

## Introduction

Fibrolamellar hepatocellular carcinoma (fHCC) is a rare type of tumor that primarily affects children and young adults. Currently, there are no reliable serum tumor markers available for clinical use (1). The production of neurotensin, a neuroendocrine marker, by fibrolamellar hepatocellular carcinomas was first reported in 1984 (2, 3). Although the molecular mechanisms have been investigated (4), detailed clinical studies have not been performed, likely due to the unavailability of routine laboratory tests.

Procalcitonin (PCT) has long been used as a marker for bacterial infections and sepsis (5). Additionally, it is useful in monitoring patients with neuroendocrine tumors (6–8). However, there are only three single-case reports on PCT-producing fHCC in the international literature (9–11).

In this report, we communicate our experience with three patients who were treated in the last 4 years at the same institution for this rare condition and demonstrate the potential usefulness of PCT as a tumor marker for follow-up assessments.

## Case descriptions

Between 2021 and 2025, the senior author (İÖ) treated five patients who were diagnosed with fHCC; all patients had increased PCT levels, although the results could not be conclusively interpreted in two of the cases. One patient underwent a major resection at another institution, which was complicated by a refractory biliary fistula; occasional bouts of cholangitis, development of recurrent tumors, and complications due to local treatments made it difficult to reach a definitive conclusion regarding the cause of the increased PCT levels. Another patient sought treatment at another unit in 2021 but rejected it; his PCT level was not measured. He returned in 2023 with obstructive jaundice caused by an enlarged tumor. His PCT level was recorded at 3.4 ng/mL (normal range < 0.05). Since he did not have cholangitis, the tumor was considered the most probable cause of the increased PCT levels. However, this could not be definitively demonstrated because only palliative care was available. The other three patients are reported below.

### Case 1

**Figure 1:**
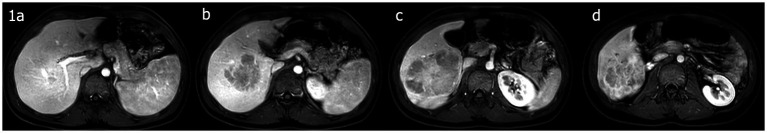
Tumor was located between the main tributaries of the right portal vein branch.

A 17-year-old boy was examined at another institution for pain in the right upper abdominal quadrant and right lower hemithoracic area. His family decided to seek treatment in our institution. His medical history and physical examination findings were unremarkable. The results of the standard blood tests were within normal limits, except for markedly increased levels of CRP (127 mg/L; normal range: 0–5) and PCT (41 ng/mL). The patient had no evidence of infection. On contrast-enhanced magnetic resonance imaging (MRI), a heterogeneous mass lesion measuring 85 × 107 mm was observed in the right lobe of the liver; it was hypervascular in the arterial phase, showed washout in the delayed phase, demonstrated diffusion restriction on diffusion-weighted imaging, and appeared hypointense in the hepatobiliary phase (Figures 1a–d). On PET-CT, a lobulated mass lesion with intense hypermetabolic FDG uptake (SUD_max_: 18.4), suggestive of malignancy, was observed. There was no evidence of extrahepatic disease.

Right hepatectomy and cholecystectomy were performed. The C-reactive protein (CRP) levels increased to 158 mg/L on postoperative day 2 (POD 2) but decreased to 61 mg/L on POD 7. The PCT levels consistently decreased to 0.6 ng/mL on POD 7. They further dropped to 0.08 ng/mL on POD 30 (CRP: 6 mg/L) and to 0.07 ng/mL on POD 88.

**Figure 2:**
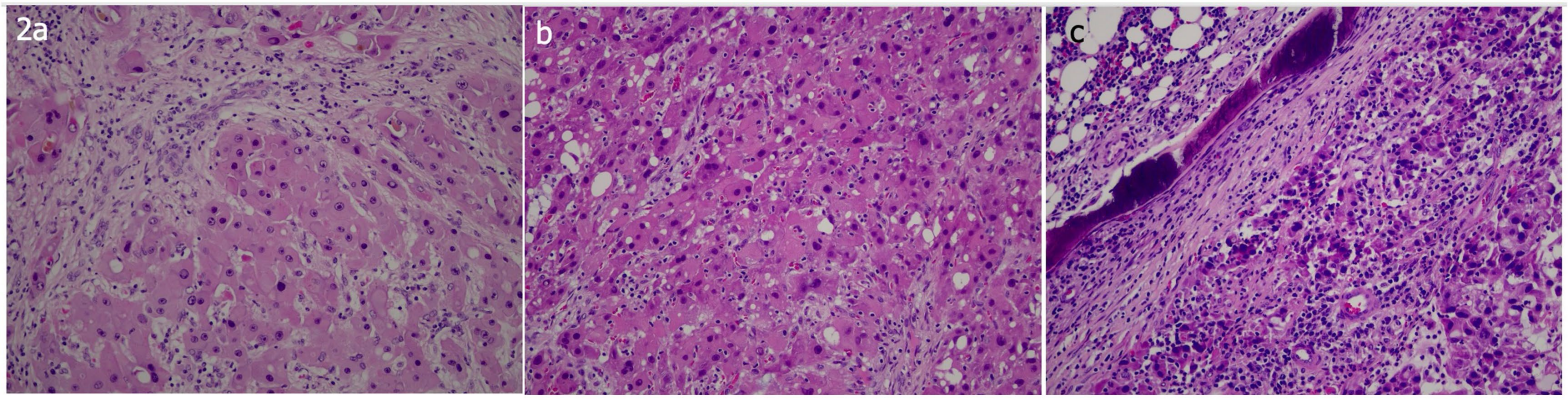
**(a)** Primary tumor, **(b)** metastasis to the thoracic wall, and **(c)** metastasis to the femur. All images are stained with hematoxylin and eosin (H&E), original magnification ×20.

Examination of the specimen revealed a solitary, well-circumscribed but unencapsulated hepatic tumor. The tumor was composed of large polygonal cells with abundant eosinophilic, with granular cytoplasm and centrally located vesicular nuclei containing prominent nucleoli. The cells were arranged in thick trabeculae and nests separated by broad, lamellar bands of hyalinized collagen. The tumor was diagnosed as moderately differentiated fibrolamellar hepatocellular carcinoma (Figure 2a). Mitotic activity was low, and there was no evidence of tumor necrosis. Only small-vessel type microscopic venous invasion was present. There was no perineural invasion. The adjacent non-tumorous liver parenchyma showed mild macrovesicular steatosis without any signs of fibrosis, hepatocellular dysplasia, chronic hepatitis, or significant iron deposition. The surgical resection margin was free of tumor, with the closest margin located 15 mm from the lesion. One regional lymph node from the gallbladder bed was negative for metastatic disease. Immunohistochemical studies revealed positive immunoreactivity to HepPar-1, CK7, and CD68. Although the tumor cells partly resembled those of neuroendocrine neoplasms, no true neuroendocrine differentiation was identified, and immunohistochemical staining for neuroendocrine markers (synaptophysin and chromogranin) remained negative.

**Figure 3:**
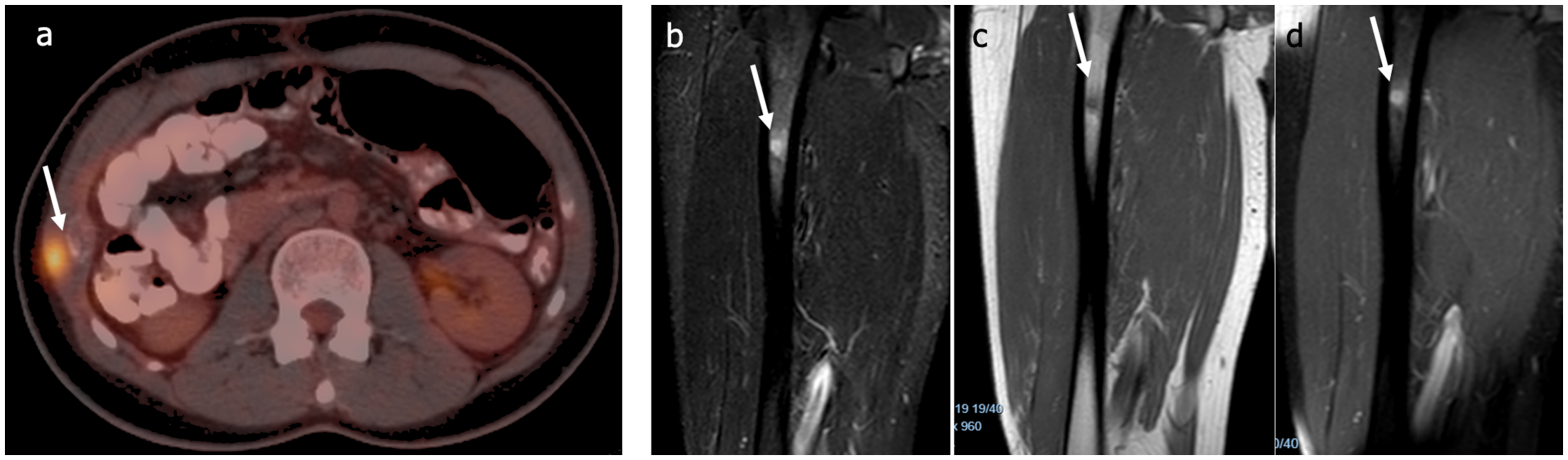
**(a)** Thoracic wall metastasis detected by PET-CT (arrow). The MRI of the femur showed an 8 mm intramedullary lesion (arrow), which was **(b)** hyperintense on T2 STIR sequences, **(c)** hypointense on T1-weighted sequences, and **(d)** enhanced contrast-enhanced T1-weighted sequences.

At 7 months, the PCT level was found to have risen from 0.07 to 0.54 ng/mL. There were no signs of recurrence on the abdominal MRI. However, PET-CT showed an expansile lytic lesion, breaching the cortical integrity on the lateral part of the right tenth costal arch (SUVmax:12.4) (Figure 3a), and a suspicious area in the proximal diaphysis region of the femur (in the medullary area). Contrast-enhanced MRI of the femur showed an 8-mm nodular, intramedullary lesion in the diaphysis (Figures 3b–d). Although both lesions were technically ‘resectable’, two early recurrences led to the selection of an initial non-operative approach, especially in light of the increase in PCT levels to 0.8 ng/mL within 10 days. Stereotactic radiotherapy was performed. The PCT level decreased to 0.37 ng/mL in the course of treatment but increased to 0.56 ng/mL 1 month after. Follow-up PET-CT showed progression of the thoracic wall lesion to 31 × 16 mm (SUD max 14); no other activity at other sites, including the right femur, was detected.

Extensive resection of the right 10th rib was performed due to metastatic disease at the end of the 11th postoperative month. Histopathologic examination showed a tumor infiltrating the bone tissue, displaying histologic and immunohistochemical features similar to those of the hepatic lesion (Figure 2b). The tumor was adjacent to the inferior soft tissue surgical margin. Three chest wall lymph nodes showed reactive hyperplasia. The PCT level decreased to 0.02 ng/mL on POD 20.

At the end of 15 months after hepatectomy, PET-CT showed potential local recurrence on the lateral side of the 9th rib. Furthermore, a markedly hypermetabolic metastatic lesion in the meta-diaphysis area and a new minimally hypermetabolic recurrent lesion at a more cranial level on the anterior cortical surface of the right femur were detected. Contrast-enhanced MRI of the femur showed that the single 8-mm lesion had increased in size to 13 mm. Since the recurrent disease was still restricted to two locations, surgery was planned. The PCT level was 0.05 ng/mL.

Segmental resection of the femur (3 cm in each direction from the tumor) was performed. All soft tissues attached to the resected tumor-bearing segment were carefully removed, and the intramedullary tumor tissue was curetted. The specimens were submitted for microbiological culture and histopathologic examination. The bone segment was subsequently immersed in a liquid nitrogen tank for 20 min, kept at room temperature for 15 min, and finally placed in a rifampicin solution for 10 min. The medullary canal was then filled with antibiotic-impregnated bone cement, and the frozen autograft was reimplanted. Histopathologic examination revealed infiltration by malignant tumor cells, resembling the primary hepatic lesion (Figure 2c), with positive immunoreactivity for HepPar-1. Both the proximal and distal surgical margins were free of tumor.

As there was a possibility for residual or recurrent malignancy on the thoracic wall, a second surgical procedure was planned. The right 9th rib and a posterior segment of the 10th rib were removed to extend the previous resection area, and the thoracic wall was reconstructed with a polypropylene mesh. Histopathologic examination showed inflammatory and regenerative changes only; there was no evidence of malignancy, which was compatible with the normal PCT level.

The patient had excellent liver function and showed no signs of chronic liver disease. Treatment was initiated with bevacizumab at a dosage of 15 mg/kg/day and atezolizumab at 1200 mg/day every 21 days. Since the alpha-fetoprotein level was normal, the treatment response had to be monitored with imaging and PCT measurements. At 42 months of follow-up, no new recurrence sites were detected on PET-CT and MRI scans, and the latest PCT level was 0.04 ng/mL.

### Case 2

A 22-year-old woman visited the emergency department of another institution for non-specific right upper abdominal pain. Ultrasonography showed a hepatic mass in the right lobe, which prompted an MRI; the images were interpreted as showing a hemangioma (51 × 50 mm), and conservative follow-up was recommended. However, she experienced sudden weight loss in the subsequent months, leading to further investigations at another institution. The PET-CT scan showed a liver mass with a SUDmax of 3.2 (slightly higher than the parenchymal level of 2.4). The MRI showed enlargement of the lesion to 59 × 53 mm (maximum dimension in the craniocaudal direction was 8.5 cm); a percutaneous biopsy confirmed the diagnosis of fHCC.

The patient presented to our center for surgery. The results of the standard blood tests were within normal limits, except for a mildly increased CRP level (21 mg/L) and a markedly increased PCT level (5.8 ng/mL). The patient had no evidence of infection. Right hepatectomy and cholecystectomy were performed. The CRP level increased to 45 mg/L on POD2, but it decreased to 13 mg/L on POD 7. The PCT level decreased consistently to 0.23 ng/mL on POD 7. On POD 35, the PCT level decreased to 0.05 ng/mL, and the CRP level dropped to 1.1 mg/L.

Histopathologic examination revealed moderately differentiated fibrolamellar hepatocellular carcinoma. Tumor necrosis was present, involving approximately 10% of the lesion. The main tumor was unifocal; however, microscopic tumor foci were also identified in several periportal areas surrounding the primary lesion. Only a small-vessel type microscopic venous invasion was present. No perineural invasion was identified. The surrounding non-tumorous liver parenchyma exhibited moderate inflammatory activity with mild fibrosis. The surgical resection margins were tumor-free. Immunohistochemical studies revealed positive immunoreactivity for HepPar-1, CK7, and CD68.

She was followed up with an MRI of the liver and a CT of the thorax as needed. She did not experience any recurrence over 16 months, and her PCT levels remained within the normal range.

### Case 3

A 24-year-old man presented to the emergency department of another institution for non-specific right upper abdominal pain. Ultrasonography and non-contrast CT results were interpreted as indicating a hemangioma in the right lobe. However, persistent complaints led to re-imaging 6 months later. The CT scan showed enlargement of the lesion, prompting a biopsy. The patient then attended our institution, where MRI findings were compatible with an fHCC. The lesion size was found to be increased from 69 × 52 mm to 94 × 67 mm. The biopsy confirmed the diagnosis of fHCC. The results of the standard blood tests were within normal limits, except for a mildly increased level of CRP (14 mg/L) and markedly increased PCT levels (1.2 ng/mL). The patient had no evidence of infection. Right hepatectomy and cholecystectomy were performed. The CRP level increased to 73 mg/L on POD 2 and fell to 56 mg/L on POD 7. The PCT level decreased consistently to 0.2 ng/mL on POD 7. The postoperative course was complicated by a Grade A biliary fistula (12). On POD 18, the PCT level decreased to 0.08 ng/mL and the CRP dropped to 7 mg/L.

Histopathologic examination of the specimen showed a moderately differentiated fibrolamellar hepatocellular carcinoma. No tumor necrosis, lymphovascular, or perineural invasion was observed. The surgical resection margin was free of tumor, with the closest margin located 1 mm from the lesion. The surrounding non-tumorous liver parenchyma showed congestion and mild lobular inflammation. Immunohistochemical studies revealed positive immunoreactivity for CK7, HepPar-1, arginase-1, and CD68.

He had no recurrence during the MRI performed at the 6-months follow-up, and the PCT levels remained within normal limits.

## Discussion

The delays in diagnosing the two patients reported here were due to a failure to interpret the available imaging data properly (case 2) and to consider the possibility of malignancy in young adults (cases 2 and 3) (13, 14). In other words, an MRI using a hepatocyte-specific agent would have been sufficient for a diagnosis.

It has been stated that “Transcriptional profiling of pure fibrolamellar hepatocellular carcinoma reveals an endocrine signature.” (15). Evidence supporting (16) and contradicting (17, 18) this notion has been reported. Still, neurotensin production by fHCC is not disputed (2–4, 19). However, there is no routinely available laboratory test for this marker. The high PCT levels in the three patients reported in detail here were incidental, i.e., PCT was a part of our workup at admission. The initial workup of an fHCC patient with an increased PCT level should exclude numerous infectious and inflammatory conditions that may have caused this finding (20). For example, there was a possibility of infection in two of our five patients. Although definite proof of PCT production by the tumor could not be obtained (anti-PCT antibody for immunohistochemistry was not available at our institution), the courses of PCT levels provide persuasive evidence. Right hepatectomy with negative surgical margins resulted in the eventual normalization of PCT levels in two patients, while one patient experienced a decrease in levels from 41 ng/mL to a near-normal level of 0.07 ng/mL. Monitoring PCT levels enabled us to detect asymptomatic bone metastases (in the rib and femur) early and initiate effective treatment in that one patient. The early (<1-year post-hepatectomy) appearance of two bone metastases initially raised the possibility of a grim outcome. There are case reports describing the successful resection of lung metastases of fHCC (21), but there is a lack of similar experiences with bone metastases. Therefore, stereotactic radiotherapy was used initially to gain time to predict the overall prognosis. Since the recurrent disease was confined to two sites, an aggressive surgical intervention was performed, and local control was achieved at both locations. Although longer follow-up is necessary for a definitive assessment, the absence of new recurrences at the end of 42 months suggests that the judicious and vigilant approach was suitable for the patient. To the best of our knowledge, this is the first reported case where an increased PCT level served as the initial indicator for the detection and treatment of recurrent disease. In the previous reports on three patients (9–11), PCT production by fHCC was confirmed by dramatic decreases in blood levels after hepatectomy in two patients (9, 11), genetic analysis in one patient (9), and immunohistochemistry in another (10).

Disease recurrence at two sites in case 1 was accompanied by a modest PCT level of 0.54 ng/mL, which may have been caused by common conditions such as upper respiratory tract infection. This observation led to the policy of contacting patients before planned follow-up and postponing appointments for 2 weeks if necessary due to prevailing circumstances.

The atezolizumab plus bevacizumab combination (22) is considered a landmark therapy in liver cancer (23). Unfortunately, fibrolamellar hepatocellular carcinoma cases were not eligible for the trials. The published experience with mixed results is limited to case reports (24, 25). Because there is no established regimen for metastatic fHCC, conventional chemotherapy, sorafenib, and immunotherapy plus vascular endothelial growth factor inhibitor (VEGF)/or checkpoint inhibitor combinations have been used (26, 27). Hepatectomy with negative surgical margins, resection of bone metastases after assessing tumor biology, and administration of immunotherapy plus VEGF combination yielded a satisfactory outcome.

Although neuroendocrine differentiation has not been shown in the three previously reported cases or in the three patients discussed in this manuscript, PCT may be useful for monitoring fHCC. These observations should be confirmed in larger studies. Since PCT measurement is routinely available, baseline levels should be measured at least once in the workup of patients with fHCC. If future results are encouraging, rapid accumulation of reliable data can be achieved by immunohistochemical examination of archived fibrolamellar hepatocellular carcinoma samples.

## Data Availability

The data analyzed in this study is subject to the following licenses/restrictions: This is a retrospective case report on three patients. More information on the patients will be provided by the corresponding author on reasonable request. Requests to access these datasets should be directed to iozden@hotmail.com.
